# The Water Supply of East London

**Published:** 1898-10-01

**Authors:** 


The Hospital

A Journal of

The Medical Sciences and Hospital Administration.

Vol. XXV.—No. 627.] Edited by Sir Henry Burdett, K.C.B., and Solomon C. Smith, M.D., M.R.C.P. [Oct. 1, 1898.

### THE WATER SUPPLY OF EAST LONDON.

It is not often that a Minister of the Crown has so complete an opportunity of exposing the hollowness of an alleged grievance as that which was afforded to Mr. Chaplin by the deputation, said to be composed of "members of East-end local authorities," but collected together and engineered by the "Social Democratic Federation," which waited upon him on the 24th instant in order to complain of a defective water supply in the district appertaining to the East London Company. The facts of the case are extremely simple. Two or three years ago the directors of the company in question became aware that their supply would be liable to fall short in the course of a prolonged dry season, and they applied to Parliament for the powers necessary to make reservoirs of sufficient size to obviate the difficulties which they anticipated. These powers were refused to them in consequence of the opposition of the London County Council, a body which at that time hoped to become the "water authority" for the whole of the metropolis, and which objected to any enhancement of the value of a plant which it might be called upon to purchase. In the following year a scarcity of water in East London proved beyond question that the directors had not over-estimated the emergency which they might be called upon to encounter; and, on the strength of the evidence thus furnished, they obtained their enabling Act without any great opposition. But a whole year had been thrown away, and the construction of reservoirs is a matter of time. Before they were completed, the prolonged drought and the almost unexampled heat of the past summer practically abolished the supply from the River Lea, and the company found themselves with a deficiency of fifteen millions of gallons daily, as compared with the quantity which they were accustomed to distribute. The only course open to them was to appeal for assistance to other companies, and, in the meanwhile, to substitute an intermittent for a constant service. Both these things were done, and done promptly; and the assistance was willingly rendered. But it could be rendered only in one way, namely, by the establishment of connections between the mains of the East London Company and those of the companies which came to its aid, except in the case of the New River Company, which was able to forego its own claims upon the River Lea, and thus to surrender a large amount of water which was suffered to flow past its own intake, and to become available for the demands of East London. This, of course, could be done upon demand, but the construction of new connections was a matter of engineering, which required time as a necessary condition of sound workmanship. In the meanwhile the East London Company was still able to give a supply for four hours daily, a supply which would have been abundant had the inhabitants of the district possessed any adequate means of storage. The real inconvenience fell upon the very poor, who had no vessels of sufficient capacity to contain water enough for the demands of the day, and by those whose cisterns were beyond the reach of the lowered pressure. The needs of the poor in this respect were speedily met—a large number of stone bottles and other receptacles being distributed. At the time of writing this the connections with the mains of other companies have been made, and the trouble seems likely to be soon at an end.

There can be no doubt that the so-called "famine" was productive of considerable inconvenience among a population accustomed to a constant supply, scantily provided with means of temporary storage, and too much in the habit of treating water as a commodity of no value, to be scattered and wasted in any conceivable quantity. More than this cannot be said. Fortunately there seems, so far, to have been no increase of mortality in the district; although it is alleged that "street smells" have been somewhat more in evidence than usual. Coleridge said that he was able to discover seventy-two distinct stinks in Cologne; and it is doubtful whether even a German town, however well intentioned, could surpass Whitechapel in this respect. The very careful examination of the streets and houses concerned, which has been made by a special correspondent of the *Times*, and published in a series of highly interesting letters, is sufficient to show that, if there were any great hardships, the inhabitants themselves were in many cases cheerfully unconscious of them. The whole matter resolved itself into a necessity for taking more care and more trouble than were usually required.

